# Implantes Potencialmente Inapropriados de Cardioversor-Desfibrilador na Prevenção Secundária de Morte

**DOI:** 10.36660/abc.20220899

**Published:** 2024-10-22

**Authors:** William Neves de Carvalho, Tainá Teixeira Viana, Clara Salles Figueiredo, Fernanda Martins, Luiz Carlos Santana Passos

**Affiliations:** 1 Universidade Federal da Bahia Salvador BA Brasil Universidade Federal da Bahia – UFBA, Salvador, BA – Brasil

**Keywords:** Desfibriladores Implantáveis, Cooperação e Adesão ao Tratamento, Adesão à Medicação, Vulnerabilidade Social, Equipe de Assistência ao Paciente

## Abstract

**Fundamento:**

Os cardioversores-desfibriladores implantáveis (CDIs) são indicados para pacientes que apresentaram taquiarritmias malignas por causas irreversíveis, clinicamente estáveis e que tenham expectativa de vida maior que um ano. No entanto, condições socioeconômicas e psicossociais desfavoráveis impactam negativamente a sobrevida de curto prazo e podem tornar o implante inapropriado.

**Objetivo:**

Avaliar se marcadores econômicos e psicossociais (MEPS) estão associados a maior mortalidade no primeiro ano (indicando implantes potencialmente inapropriados) após implante do CDI.

**Métodos:**

Coorte prospectiva entre 2017 e 2021 incluindo pacientes com insuficiência cardíaca com fração de ejeção do ventrículo esquerdo (FEVE) < 50% submetidos a implante de CDI como profilaxia secundária. Antes do procedimento, foram avaliados por uma EMD que investigou quatro variáveis denominadas MEPS: vulnerabilidade socioeconômica, capacidade do autocuidado, adesão farmacológica e transtornos do humor. Os participantes foram acompanhados por no mínimo 12 meses. Foi considerado significância estatística valores-p < 0,05.

**Resultados:**

Foram incluídos 208 indivíduos, sendo 144 (68,9%) do sexo masculino. A FEVE média foi 32% ±9 e 107 (51%) tinham etiologia chagásica. A mortalidade no primeiro ano foi 54/208 (25,8%). Todos os pacientes que faleceram tinham ao menos um dos MEPS e não houve óbitos entre os 73 (35,4%) que não tinham MEPS. Em análise multivariada ter MEPS e a FEVE foram os únicos preditor independente na mortalidade menor que 1 ano: RR 20,48 (2,75 – 52,29); p=0,003 e RR 0,97 (0,93 – 0,99); p=0,047, respectivamente.

**Conclusão:**

Condições socioeconômicas e psicossociais devem ser identificadas e quando possível resolvidas antes do implante, pois podem tornar o implante do dispositivo um procedimento potencialmente inapropriado.

**Figure:**
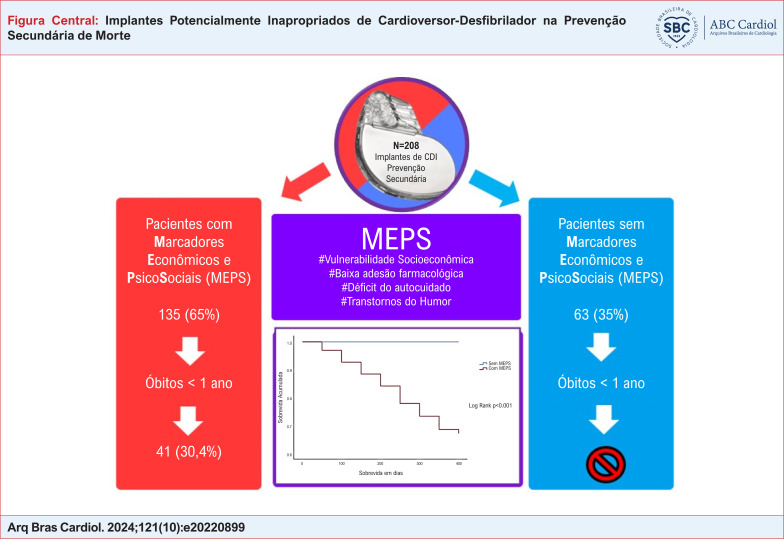


## Introdução

O cardioversor-desfibrilador implantável (CDI) é a terapia indicada para prevenção secundária de morte súbita cardíaca (MSC) em pacientes ressuscitados de parada cardiorrespiratória por taquicardia ventricular (TV), fibrilação ventricular (FV), eventos sincopais recorrentes de origem arritmogênica e eventos de TV com instabilidade hemodinâmica, incluindo, em grande parte, pacientes com insuficiência cardíaca com fração de ejeção reduzida (ICFER).^1,2^ Os candidatos elegíveis a essa terapia devem ter expectativa de vida superior a 1 ano e estarem em boas condições clínicas para realizar o procedimento.^1,2^ Apesar da sua eficácia comprovada em médio e longo prazo, alguns estudos têm demonstrado resultados questionáveis da eficácia do CDI na redução de mortalidade em determinadas populações que apresentam condições socioeconômicas e psicossociais precárias que impactam a sobrevida em curto prazo e podem tornar o implante do CDI uma terapia inapropriada.^3–11^

A ICFER é uma doença que está associada à alta incidência de outras comorbidades que deixam os pacientes fragilizados.^12,13^ Com isso, muitos candidatos elegíveis citados nas diretrizes podem, além das comorbidades, apresentar situações de vulnerabilidade socioeconômica, negligência e incapacidade do autocuidado, dificuldades na adesão ao melhor tratamento farmacológico e, com frequência, distúrbios psicológicos como ansiedade e depressão. Esses marcadores econômicos e psicossociais (MEPS) são questões particularmente relevantes em países em desenvolvimento, onde o acesso aos serviços de saúde é limitado.^1,6,7,10,11,14–16^

Diante da mortalidade elevada por diversas causas associadas à ICFER, uma estratégia altamente recomendada pelas diretrizes de IC é a inclusão de equipe multidisciplinar (EMD) para avaliar de forma mais criteriosa e integral. Em casos mais complexos, fomentar discussões em programas chamados *"Heart Team",* onde ocorrem as tomadas de decisão das melhores condutas e terapias disponíveis.^1,17–19^

Nesse contexto, além de médicos, é necessário envolver outros profissionais (enfermeiros, psicólogos, assistentes sociais, nutricionistas e farmacêuticos) na avaliação antes do implante. Essa abordagem tem como objetivo identificar indivíduos com critério de indicação para implante, mas apresentam variáveis menos óbvias, que não são do domínio ou interesse dos especialistas em dispositivos cardíacos eletrônicos implantáveis (DCEI), mas que podem ser preditoras de mortalidade precoce.^1,19^

Sendo assim, o objetivo do presente estudo é verificar a associação entre a presença de MEPS desfavoráveis e a mortalidade por todas as causas no primeiro ano (indicando implantes potencialmente inapropriados) nos indivíduos que foram avaliados por uma EMD e submetidos a implante de CDI como prevenção secundária de morte.

## Métodos

### Desenho do estudo

Trata-se de uma coorte observacional e prospectiva de um único centro na Região Nordeste do Brasil que ocorreu no período entre 2017 e julho de 2021. O centro do estudo é o principal hospital referência no tratamento de doenças cardiovasculares e o único a realizar implantes de CDI pelo Sistema Único de Saúde (SUS) no Estado da Bahia.

### População do estudo

Os requisitos de elegibilidade incluíram pacientes ≥ 18 anos com IC FEVE <50%, com indicação de implante de CDI como prevenção secundária de MSC conforme as diretrizes. Os eventos considerados como prevenção secundária foram os indivíduos ressuscitados de parada cardiorespiratória (PCR) em ritmo de TV/FV ou aqueles que apresentaram TV sintomática. Os indivíduos deveriam ter expectativa de vida maior que um ano.^2,15^ A EMD foi formada por profissionais médicos e não médicos (enfermeiros, nutricionistas, psicólogos, farmacêuticos, assistentes sociais e cardiologistas clínicos). Após coleta de dados (aplicação dos instrumentos), os casos foram levados para discussão em um "*Heart Team*" específico, denominado institucionalmente de "*Device Team (DT)*, formado exclusivamente para casos dos candidatos a implante de DCEI de alto custo.^19^

### Desfecho

O desfecho primário foi mortalidade em período inferior a um ano após o implante do dispositivo (considerados implantes potencialmente inapropriados).

Todos os pacientes foram monitorados por no mínimo 12 meses, sendo por telefone a cada 30 dias e presencialmente a cada 6 meses, de acordo com normas da instituição.

### Variáveis de interesse

Após a indicação formal do implante do CDI por um arritmologista, todos os pacientes foram encaminhados para entrevista presencial com profissionais de uma EMD composta por um enfermeiro especialista em insuficiência cardíaca (IC), um cardiologista clínico, um psicólogo, um farmacêutico e um assistente social. Foram coletadas informações sobre dados clínicos como NYHA, presença de comorbidades prévias, uso prévio de medicação para tratamento de IC e controle de arritmias, dados antropométricos, exames laboratoriais e ecocardiográficos, além da avaliação de fatores psicossociais e socioeconômicos: transtornos de humor, adesão medicamentosa, capacidade do autocuidado, renda familiar e escolaridade.

Os exames laboratoriais foram realizados até 24 horas antes do implante. A taxa de filtração glomerular foi avaliada por CKD-EPI. Todos os exames de ecocardiograma foram realizados até 6 meses antes do procedimento. Foi considerada IC com fração de ejeção reduzida quando FEVE <50% pelo método de Simpson bidimensional.

Os transtornos de humor foram avaliados por meio da escala hospitalar de ansiedade e depressão (HADS-A e HADS-D), e quando a soma dos pontos da escala foi maior que 8 (HADS-A> 8 ou HADS-D> 8) foi considerado com algum grau de desordem.^5^ A baixa adesão farmacológica foi medida pela escala de *Morisky*, e aqueles com < 6 pontos foram considerados de baixa adesão.^14^ A autonomia e capacidade de manutenção do autocuidado foi avaliada por meio do questionário *Self Care Hf*. Aqueles com menos de 70 pontos foram considerados com déficit de autocuidado.^12^ Consideramos vulnerabilidade socioeconômica os pacientes que autorrelataram renda familiar menor que um salário-mínimo e baixa escolaridade (analfabetos ou com até 5 anos de escolaridade).

### Análise estatística

O teste de Kolmogorov–Smirnov foi utilizado para verificar a distribuição normal das variáveis contínuas. Variáveis com distribuição normal foram descritas por médias e desvios-padrão e comparadas pelo teste T de Student não pareado. Variáveis com distribuição não normal foram descritas pelas medianas e intervalos interquartis 25% e 75% e comparadas pelo teste de Mann-Whitney. As variáveis categóricas foram descritas como frequências e porcentagens e comparadas pelo teste do qui-quadrado. A curva de Kaplan-Meier foi usada para estimar a sobrevida e o teste Log-Rank foi usado para comparar a sobrevida entre os grupos. Para análise multivariada, foi utilizado o modelo de Cox, incluindo variáveis com possível associação com o desfecho (p<0,1). Um valor-p <0,05 foi considerado estatisticamente significativo. O *Statistical Package for the Social Sciences* (SPSS) versão 20.0 foi utilizado para a análise de todos os dados.

De acordo com a resolução 466/2012 do Conselho Nacional de Saúde, o presente estudo foi aprovado pelo comitê de ética em pesquisa local e todos os procedimentos foram realizados de acordo com a Declaração de Helsinque.

## Resultados

Foram incluídos 208 indivíduos submetidos a implante de CDI para profilaxia secundária de morte súbita. Destes, 144 (68,9%) eram do sexo masculino, a idade média foi de 58 ±12 anos, 91 (43,5%) estavam em classe funcional NYHA III, e a FEVE média foi de 32%±9. A cardiopatia chagásica foi a etiologia mais prevalente, representando 107 (51%) pacientes, seguida da cardiopatia isquêmica, com 52 (25%) pacientes (Tabela 1). A média da estimativa de mortalidade em 1 ano pelo Maggic score foi de 15,9±10,5%.

**Tabela 1:** Características basais

	Total n=208	Com MEPS	Sem MEPS
Masculino; n (%)	144 (68,9%)	92 (68,1%)	52 (70,3%)
Idade, média (±DP)	58,1 (±12,3)	59,9 (±12,3)	54,9 (±11,8)
Hipertensão, n (%)	154 (73,7%)	104 (77%)	50 (67,6%)
Diabetes, n (%)	52 (24,9%)	40 (29,6%)	12 (16,2%)
Fibrilação atrial, n (%)	51 (24,4%)	41 (30,4%)	10 (13,5%)
AVC prévio, n (%)	29 (13,9%)	22 (16,3%)	7 (9,5%)
Infarto do miocárdio prévio, n (%)	66 (31,6%)	47 (37,4%)	19 (25,7%)
Doença renal crônica, n (%)	36 (17,2%)	28 (20,7%)	8 (10,8%)
FEVE, média (±DP)	32,1 (±9,3)	31,5 (±9,2)	33,0 (±9,6)
Ressuscitado de PCR em FV/TV, n (%)	74 (35,4%)	50 (37,0%)	24 (32,4%)
NYHA III, n (%)	91 (43,5%)	76 (54,3%)	15 (20,3%)
Miocardiopatia chagásica, n (%)	107 (51,2%)	34 (45,9%)	73 (54,1%)
Miocardiopatia isquêmica, n (%)	52 (24,9%)	19 (25,7%)	47 (34,8%)
iECA ou BRA ou INRA, n (%)	151 (72,2%)	95 (70,4%)	56 (75,7%)
Betabloqueadores, n (%)	163 (78,0%)	101 (74,8%)	62 (83,4%)
Amiodarona, n (%)	120 (57,4)	84 (62,2%)	36 (48,6%)
MAGGIC score%, média (±DP)	15,9 (±10,5)	18,0 (±111,5)	12,0 (±6,9)

AVC: acidente vascular cerebral; IMC: índice de massa corporal; iECA: inibidores da enzima conversora da angiotensina; BRA: bloqueador do receptor da aldosterona; INRA: inibidores do receptor da neprilisina; FEVE: fração de ejeção do ventrículo esquerdo; MEPS: marcadores econômicos e psicossociais; NYHA: New York Heart Association; TV: taquicardia ventricular; FV: fibrilação ventricular.

O tempo médio de seguimento foi de 586 ±407 dias, sem perda de seguimento. As indicações para o implante do dispositivo foi TVS sintomática em 135 (64,6%) indivíduos e PCR em ritmo de FV/TV em 74 (35,4%). No primeiro ano pós implante do CDI, a mortalidade geral foi de 54/208 (25,8%) e, ao longo de todo período do estudo, foi de 41/208 (19,6%) óbitos.

A EMD identificou pelo menos um dos MEPS em 135 (64,6%) indivíduos. Os MEPS mais prevalentes foram: vulnerabilidade socioeconômica (48,8%) e déficit no autocuidado (39,7%).

**Figura 1:**
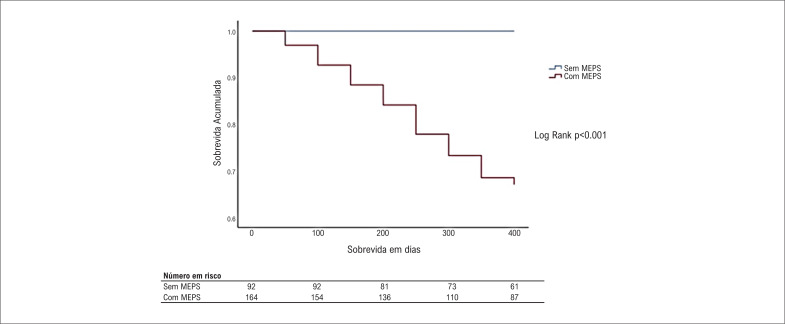
Curva de sobrevida (Kaplan-Meier) em um ano de pacientes após implante de CDI como prevenção secundária realizados s no Hospital Ana Nery entre 2017 e 2021. MEPS: marcadores econômicos e psicossociais.

Não houve óbito no período menor que um ano dentre os pacientes sem MEPS, enquanto nos pacientes que apresentavam pelo menos 1 MEPS, a mortalidade no mesmo período foi 41 (30,4%), p<0,001 (Figura 1).

**Figura 2:**
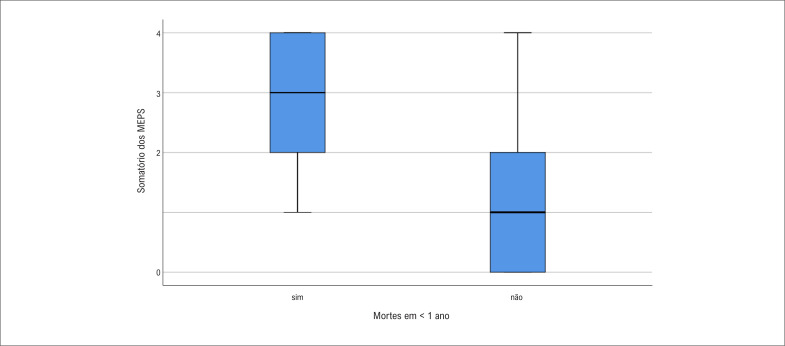
Quantidade de marcadores econômicos e psicossociais (MEPS) desfavoráveis e sua relação com mortalidade no primeiro ano após implante de CDI.

O boxplot (Figura 2) mostra a distribuição dos dados referentes à frequência de MEPS presente em cada paciente e à mortalidade em um ano. A mediana de fatores entre os que evoluíram a óbito foi de 3 (1-4), enquanto foi de 1 (0-2) entre os que estavam vivos ao final do primeiro ano (p<0,001).

A Tabela 2 mostra a comparação univariada das variáveis clínicas e psicossociais entre os indivíduos que evoluíram a óbito e os sobreviventes. Na análise multivariada, incluindo fatores clínicos que apresentaram associação na univariada ou com plausibilidade biológica de associação com o desfecho primário, apenas a presença dos MEPS e a FEVE foram preditores independentes da mortalidade em um ano: HR 20,48 (2,75 – 52,29); p=0,003 e HR 0,97 (0,93 – 0,99); p=0,047, respectivamente (Tabela 3).

**Tabela 2:** Comparação das características basais dos indivíduos sobreviventes e os que evoluíram a óbito no primeiro ano após implante

	Sobreviventes	Óbitos	p
	167 (80,3%)	41 (19,7%)	
Masculino; n(%)	112 (67,1%)	31 (75,6%)	0,349
Idade, média (±DP)	57 (±12)	62 (±12)	0,040
Miocardiopatia chagásica, n(%)	86 (51,5%)	21 (51,2%)	0,999
Miocardiopatia isquêmica, n(%)	40 (24%)	12 (29,3%)	0,546
Ouras etiologias conhecidas, n(%)	52 (25,5%)	10 (19,2%)	0,307
Hipertensão Arterial Sistêmica, n(%)	123 (73,7%)	30 (73,2%)	0,999
Diabetes, n(%)	38 (22,8%)	14 (34,1%)	0,159
Fibrilação atrial, n(%)	37 (22,2%)	14 (34,1%)	0,155
Acidente Vascular Cerebral prévio n (%)	21 (12,6%)	8 (19,5%)	0,312
Infarto do miocárdio prévio, n (%)	47 (28,1%)	19 (46,3%)	0,038
Doença Renal Crônica, n (%)	26 (15,1%)	10 (24,4%)	0,247
FEVE %, média (±DP)	32,8 (±9,5)	28,9 (±8,2)	0,013
NYHA III, n (%)	64 (38,3%)	26 (63,4%)	0,005
MAGGIC score (% de risco em 1 ano), média (±DP)	14,4 (±9,1)	22,2 (±13,2)	0,001
Vulnerabilidade socioeconômica, n (%)	70 (41,9%)	32 (78,0%)	<0,001
Déficit no autocuidado, n (%)	59 (35,3%)	24 (58,5%)	0,008
Baixa adesão farmacológica, n (%)	31 (18,6%)	16 (39,0%)	0,011
Transtornos do Humor, n (%)	58 (34,7%)	24 (58,5%)	0,007
PCR em FV/ TV	57 (34,1%)	16 (39,0%)	0,586

FEVE: fração de ejeção do ventrículo esquerdo; NYHA: New York Heart Association. Transtornos do Humor: ansiedade e/ou depressão; Déficit do autocuidado: Self-care < 70 pontos; Baixa adesão farmacológica: Morisky < 6 pontos.

**Tabela 3:** Análise univariada e multivariada na mortalidade inferior a um ano após implante de CDI

	Análise	p	Análise	p
	Univariada		Multivariada	
	RR (IC de 95%)		HR (IC de 95%)	
Presença de MEPS	1,44 (1,29 – 1,61)	< 0,001	20,48 (2,75 – 52,29)	0,003
FEVE	0,96 (0,95 – 0,97)	<0,001	0,97 (0,93 – 0,99)	0,047
Doença de Chagas	1,01 (0,51 – 2,00)	0,999	1,12 (0,52 – 2,40)	0,773
NYHA III	2,27 (1,28 – 4,03)	0,005	0,99 (0,51 – 1,94)	0,978
Idade	0,98 (0,98 – 0,99)	<0,001	1,01 (0,98 – 1,04)	0,423
IAM prévio	1,86 (1,08 – 3,19)	0,038	1,86 (0,85 – 4,05)	0,120

MEPS: marcadores econômicos e psicossociais; FEVE: fração de ejeção do ventrículo esquerdo; IAM: infarto agudo do miocárdio.

## Discussão

Os fatores psicossociais e socioeconômicos estiveram associados de forma independente com maior mortalidade na população de pacientes com IC submetidos a implante de CDI para prevenção secundária de morte súbita. O que demonstra que, além da fragilidade clínica provocada pela doença, aqueles que apresentam baixa adesão farmacológica, déficit no autocuidado, vulnerabilidade socioeconômica e transtornos do humor, os MEPS, têm maior probabilidade de morte precoce.

Poucos estudos na literatura avaliaram a relação dos fatores psicológicos e socioeconômicos com o prognóstico dos indivíduos com IC ou candidatos a implante de DCEIs de alto custo.^4–10,19^

A estratégia de avaliação dos candidatos a implante de DCEI, ou pelo menos a sua sistematização horizontalizada para tomada de decisão, é pioneira no Brasil e mundialmente inédita para casos de implante de DCEIs. Dessa forma, a EMD foi fundamental para que fossem identificados importantes preditores que, em nosso seguimento, foram determinantes na mortalidade em 12 meses. Um modelo semelhante ao nosso ocorre num hospital no Reino unido, onde 80% dos tratamentos de câncer são decididos após reunião com profissionais de diferentes disciplinas de saúde.^20^

A relação entre mortalidade precoce e presença de MEPS provavelmente advém de diversos mecanismos, sendo os mais importantes levantados na literatura relacionados ao baixo entendimento dos pacientes referente ao processo de adoecimento, a falta de autonomia ou incapacidade para o autocuidado e descuido na adesão farmacológica associado a dificuldades no acesso aos recursos de saúde.^10,11,18^

O déficit no autocuidado está relacionamento com o gerenciamento inadequado de práticas e ações propositivas em relação a diminuição da ingestão hidro salina, uso irregular de medicamentos, cessação do tabagismo, não vacinação para pneumonia e influenza, bem como o monitoramento sistemático do peso. Estudos apontam que o autocuidado é deficiente na maioria dos portadores de IC com variações de acordo com fatores sociodemográficos, econômicos, cultural, educacional, etiológico, como também varia em diferentes nacionalidades.^6,21,22^

Estima-se que em torno de 60% dos pacientes com IC apresentam algum grau de ansiedade e/ou depressão e apenas um terço são diagnosticados.^4,5^ São pacientes com altas taxas de re-hospitalização e mortalidade precoce, além de experimentarem sentimentos, sensações e eventos que afetam a qualidade de vida como dificuldades de adaptação com o dispositivo, maiores chances de infecção e choques inapropriados.^4,23^ Nossa taxa de pacientes identificados com transtornos do humor foi 39,8% e tiveram impacto na mortalidade (p=0,012).

As taxas de pacientes com uso inadequado ou não sistemático dos medicamentos varia entre 30-60%. É um problema multifatorial que está associado à polifarmácia, às dificuldades socioeconômicas, à intolerância e ao acesso às drogas.^7^ Um estudo com 557 pacientes avaliou o impacto da adesão medicamentosa de pacientes com IC, um dos desfechos foi morte por qualquer causa. Após 1,1 ano de seguimento a mortalidade foi 28,5%, e aqueles com baixa adesão esteve associada isoladamente na mortalidade e com risco duas vezes maior de morte, p<0,001.^24^ Em nossa amostra apesar de ser o marcador com menor prevalência – 47/208 (22,5%), esteve associada com mortalidade no primeiro ano do implante (p=0,011).

Um dado relevante foi a elevada prevalência de indivíduos com miocardiopatia por Doença de Chagas. Este fato poderia ter relação com uma população de maior vulnerabilidade psicossocial. No entanto, o diagnóstico de miocardiopatia chagásica não apresentou associação com maior mortalidade na análise univariada ou multivariada. Portanto, este estudo levanta a hipótese de que a maior morbimortalidade relacionada à cardiopatia por Doença de Chagas pode estar ligada a um fator biológico, relacionado à infecção e seus mecanismos de lesão ao miócito, ou ser um reflexo das piores condições psicossociais a que estes indivíduos estão sujeitos, uma vez que a Doença de Chagas é mais prevalente em populações de baixa renda e com condições sanitárias precárias, devido à sua forma de transmissão.^3,10,17^

A mortalidade em um ano estimada pelo Maggic score foi de cerca de 15%; no entanto, a mortalidade identificada neste período foi de 25%. Porém, no grupo que não apresentava marcadores econômicos e sociais desfavoráveis, não houve óbito no período de um ano. Sendo assim, a taxa de mortalidade elevada desta população pode estar relacionada ao impacto destes marcadores em uma população atendida exclusivamente pelo sistema público de saúde e, portanto, com maior prevalência deles. Estudo publicado por Yusuf et al. demonstra que os fatores socioeconômicos tendem a ter maior impacto na morbimortalidade das doenças cardiovasculares quanto menor é o status socioeconômico da população.^25^

O estudo apresenta como limitações o fato de ser unicêntrico e incluir uma população com elevada prevalência de marcadores socioeconômicos desfavoráveis, porém, trata-se de uma população habitual atendida em um serviço de referência do sistema único de saúde.

Demonstramos, portanto, que tanto fatores psicossociais quanto os socioeconômicos estão interrelacionados e têm impacto direto e de maneira tão relevante quanto os fatores diretamente relacionados à biologia da doença. Sendo assim, é importante enfatizar a avaliação multidisciplinar com foco na identificação de outros agravos potencialmente resolvíveis como as doenças psicológicas, déficit no autocuidado e adesão medicamentosa antes da realização do implante do CDI, especialmente em sistemas de saúde pública, em que há escassez de recursos. Além disso, é importante investir em fatores não diretamente relacionados à saúde, mas que impactam os desfechos das políticas à saúde.

## Conclusão

A presença de fatores psicossociais e socioeconômicos desfavoráveis em indivíduos com IC submetidos a implante de CDI para profilaxia secundária de morte súbita esteve associada com um aumento de mortalidade no primeiro ano, ou seja, implantes considerados inapropriados pelas atuais diretrizes.

Sendo assim, prioritariamente deve ser preconizada a inclusão de EMD na avaliação e nas discussões do *Device Team*.
