# Supplementary material for: Implantes Potencialmente Inapropriados de Cardioversor-Desfibrilador na Prevenção Secundária de Morte
Source: Arq Bras Cardiol. 2024 Oct 22;121(10):e20220899. [Article in Portuguese] doi: 10.36660/abc.20220899 (PMC11634201; doi:10.36660/abc.20220899)
Supplement: Translation

# Translation: Potentially Inappropriate Cardioverter Defibrillator Implants in Secondary Prevention of Death

**DOI:** 10.36660/abc.20220899i

**Authors:** William Neves de Carvalho, Tainá Teixeira Viana, Clara Salles Figueiredo, Fernanda Martins, Luiz Carlos Santana Passos

**Affiliations:** 1 Salvador BA Brazil Universidade Federal da Bahia – UFBA, Salvador, BA – Brazil

**Keywords:** Implantable Defibrillators, Treatment Adherence and Compliance, Medication Adherence, Social Vulnerability, Patient Care Team

## Abstract

**Background:**

Implantable cardioverter defibrillators (ICDs) are recommended for patients experiencing malignant tachyarrhythmias due to irreversible causes, who are clinically stable, and have a life expectancy exceeding one year. However, adverse socioeconomic and psychosocial conditions can adversely affect short-term survival and may render implantation inappropriate.

**Objective:**

To assess whether economic and psychosocial markers (EPSM) are associated with higher mortality in the first year (indicating potentially inappropriate implants) following ICD implantation.

**Methods:**

A prospective cohort study conducted between 2017 and 2021 included patients with heart failure and left ventricular ejection fraction (LVEF) < 50% who underwent ICD implantation for secondary prophylaxis. Prior to the procedure, patients were evaluated by an MDT, which examined four EPSM variables, namely socioeconomic vulnerability, self-care capacity, pharmacological adherence, and mood disorders. The participants were monitored for at least 12 months. Statistical significance was considered to be p-values < 0.05.

**Results:**

A total of 208 individuals were included, with 144 (68.9%) being male. The mean LVEF was 32% ±9 and 107 (51%) had Chagas disease etiology. The mortality rate in the first year was 54/208 (25.8%). All patients who died had at least one of the EPSM and there no deaths were reported among the 73 (35.4%) who did not have EPSM. In multivariate analysis, having EPSM and LVEF were the only independent predictors of mortality under 1 year: RR 20.48 (2.75 – 52.29); p=0.003 and RR 0.97 (0.93 – 0.99); p=0.047, respectively.

**Conclusion:**

Socioeconomic and psychosocial conditions should be identified and, where possible, resolved before implantation, as they may make device implantation a potentially inappropriate procedure.

## Introduction

The implantable cardioverter defibrillator (ICD) is the therapy of choice for secondary prevention of sudden cardiac death (SCD) in patients resuscitated from cardiorespiratory arrest due to ventricular tachycardia (VT), ventricular fibrillation (VF), recurrent syncopal events of arrhythmogenic origin and VT events with hemodynamic instability, including mostly patients with heart failure with reduced ejection fraction (HFREF).^1,2^ Candidates for this therapy must have a life expectancy of more than 1 year and be in good clinical condition to undergo the procedure.^1,2^ Despite its proven efficacy in the medium and long term, some studies have shown questionable results regarding the efficacy of ICDs in reducing mortality in certain populations with precarious socioeconomic and psychosocial conditions, which impact short-term survival and may make ICD implantation an inappropriate therapy.^3–11^

HFREF is associated with a high incidence of other comorbidities that leave patients vulnerable.^12,13^ As a result, many eligible candidates described in the guidelines may have additional challenges such as socioeconomic vulnerability, neglect, inability to self-care, difficulties with medication adherence, and often psychological disorders like anxiety and depression. These economic and psychosocial markers (EPSM) are particularly relevant issues in developing countries, where access to health services is limited.^1,6,7,10,11,14–16^

Given the high mortality rates associated with HFREF from various causes, guidelines strongly recommend employing a multidisciplinary team (MDT) to assess patients comprehensively. In more complex cases, discussions within "Heart Teams" are encouraged to determine the most suitable procedures and therapies available.^1,17–19^

In this context, alongside physicians, it is crucial to involve a range of professionals (including nurses, psychologists, social workers, nutritionists, and pharmacists) during assessments before implantation. This approach aims to identify individuals who meet implantation criteria but may exhibit less apparent variables, which are not typically within the expertise of cardiac implantable electronic device (CIED) specialists but could predict early mortality.^1,19^

Therefore, this study aims to investigate the association between unfavorable EPSM and one-year all-cause mortality (indicating potentially inappropriate implants) in individuals evaluated by an MDT and who underwent ICD implantation for secondary prevention of death.

## Methods

### Study design

This is an observational and prospective cohort study from a single center in the Northeast Region of Brazil, conducted between 2017 and July 2021. The study center is the main referral hospital for the treatment of cardiovascular diseases and the only one to perform ICD implantations through the Brazilian Unified Health System (SUS) in the State of Bahia.

### Study population

Eligibility requirements included patients ≥ 18 years old with LVEF <50%, with an indication for ICD implantation as secondary prevention of sudden cardiac death (SCD) according to the guidelines. Events considered as secondary prevention were resuscitation from cardiopulmonary arrest (CPA) in VT/VF rhythm or those who presented symptomatic VT. Individuals should have a life expectancy greater than one year.^2,15^ The MDT comprised both medical and non-medical professionals (nurses, nutritionists, psychologists, pharmacists, social workers, and clinical cardiologists). Following data collection (via assessment tools), cases were discussed within a specific Heart Team designated as the Device Team (DT) for high-cost CIED implantation candidates within the institution.^19^

### Endpoint

The primary endpoint was mortality within a period of less than one year after device implantation (implants considered potentially inappropriate).

All patients were monitored for at least 12 months, by telephone every 30 days and in person every 6 months, according to the institution's standards.

### Variables of Interest

After receiving formal indication for ICD implantation from an arrhythmologist, all patients underwent a face-to-face interview with a MDT consisting of a heart failure (HF) specialized nurse, a clinical cardiologist, a psychologist, a pharmacist, and a social worker. During these interviews, information was gathered on various clinical aspects including NYHA classification, presence of pre-existing comorbidities, prior medication usage for HF management and arrhythmia control, anthropometric measurements, laboratory results, and echocardiographic findings. Additionally, the assessment covered psychosocial and socioeconomic factors such as mood disorders, medication adherence, self-care abilities, family income, and education level.

Laboratory tests were performed up to 24 hours before implantation. CKD-EPI assessed the glomerular filtration rate. All echocardiogram tests were performed up to 6 months before the procedure. HF was considered with reduced ejection fraction when LVEF <50% by the two-dimensional Simpson method.

Mood disorders were assessed using the Hospital Anxiety and Depression Scale (HADS-A and HADS-D), and the patient was considered to have some degree of disorder when the sum of the scale points was greater than 8 (HADS-A> 8 or HADS-D> 8).^5^ Poor pharmacological adherence was measured by the Morisky scale and those with <6 points were considered to have low adherence.^14^ Autonomy and ability to maintain self-care were assessed using the Self Care HF questionnaire. Those with less than 70 points were considered to have a self-care deficit.^12^ We considered patients who self-reported family income of less than one minimum wage and low education (illiterate or with up to 5 years of schooling) to be socioeconomically vulnerable.

### Statistical analysis

The Kolmogorov–Smirnov test was used to verify the normal distribution of continuous variables. Variables with normal distribution were described by means and standard deviations and compared using the unpaired Student's t-test. Variables with non-normal distribution were described by medians and 25% and 75% interquartile ranges and compared using the Mann-Whitney test. Categorical variables were described as frequencies and percentages, and compared using the chi-square test. The Kaplan-Meier curve was used to estimate survival and the Log-Rank test was used to compare survival between groups. For multivariate analysis, the Cox model was applied, including variables with a possible association with the endpoint (p<0.1). P-values <0.05 were considered statistically significant. The Statistical Package for the Social Sciences (SPSS) version 20.0 was used for the analysis of all data.

In accordance with resolution 466/2012 of the National Health Council, this study was approved by the local research ethics committee, and all procedures were performed in accordance with the Declaration of Helsinki.

## Results

A total of 208 individuals who underwent ICD implantation for secondary prophylaxis of sudden death were included. Of these, 144 (68.9%) were male, the mean age was 58 ± 12 years, 91 (43.5%) were in NYHA functional class III, and the mean LVEF was 32% ± 9. Chagas heart disease was the most prevalent etiology, accounting for 107 (51%) patients, followed by ischemic heart disease, with 52 (25%) patients (Table 1). The mean mortality estimate at 1 year by the Maggic score was 15.9 ± 10.5%.

**Table 1:** Baseline characteristics

	Total n=208	With EPSM	Without EPSM
Male; n (%)	144 (68.9%)	92 (68.1%)	52 (70.3%)
Age, average (±SD)	58.1 (± 12.3)	59.9 (± 12.3)	54.9 (± 11.8)
Hypertension, n (%)	154 (73.7%)	104 (77%)	50 (67.6%)
Diabetes, n (%)	52 (24.9%)	40 (29.6%)	12 (16.2%)
Atrial fibrillation, n (%)	51 (24.4%)	41 (30.4%)	10 (13.5%)
Previous stroke, n (%)	29 (13.9%)	22 (16.3%)	7 (9.5%)
Previous myocardial infarction, n (%)	66 (31.6%)	47 (37.4%)	19 (25.7%)
Chronic kidney disease, n (%)	36 (17.2%)	28 (20.7%)	8 (10.8%)
LVEF, mean (±SD)	32.1 (± 9.3)	31.5 (± 9.2)	33.0 (± 9.6)
Resuscitated from cardiac arrest in VF/VT, n (%)	74 (35.4%)	50 (37.0%)	24 (32.4%)
NYHA III, n (%)	91 (43.5%)	76 (54.3%)	15 (20.3%)
Chagas cardiomyopathy, n (%)	107 (51.2%)	34 (45.9%)	73 (54.1%)
Ischemic cardiomyopathy, n (%)	52 (24.9%)	19 (25.7%)	47 (34.8%)
ACEI or ARB or ARNI, n (%)	151 (72.2%)	95 (70.4%)	56 (75.7%)
Beta blockers, n (%)	163 (78.0%)	101 (74.8%)	62 (83.4%)
Amiodarone, n (%)	120 (57.4)	84 (62.2%)	36 (48.6%)
MAGGIC score%, mean (±SD)	15.9 (± 10.5)	18.0 (± 111.5)	12.0 (± 6.9)

BMI: body mass index; ACEI: angiotensin-converting enzyme inhibitors; ARB: aldosterone receptor blocker; ARNI: neprilysin receptor inhibitors; EPSM: economic and psychosocial markers; LVEF: left ventricular ejection fraction; NYHA: New York Heart Association; VT: ventricular tachycardia; VF: ventricular fibrillation.

The mean follow-up time was 586 ± 407 days, with no loss to follow-up. The indications for device implantation were symptomatic SVT in 135 (64.6%) individuals and CPA in VF/VT rhythm in 74 (35.4%). In the first year after ICD implantation, overall mortality was 54/208 (25.8%), and throughout the entire study period, 41/208 (19.6%) deaths occurred.

The MDT identified at least one of the EPSM in 135 (64.6%) individuals. The most prevalent EPSM were socioeconomic vulnerability (48.8%) and self-care deficit (39.7%).

No deaths were reported in the period of less than one year among patients without EPSM, while in patients who had at least 1 EPSM, mortality in the same period was 41 (30.4%), p <0.001 (Figure 1).

**Figure 1:** One-year survival curve (Kaplan-Meier) of patients after ICD implantation as secondary prevention performed at Hospital Ana Nery between 2017 and 2021. EPSM: economic and psychosocial markers.

The boxplot (Figure 2) shows the distribution of data regarding the frequency of EPSM for each patient and mortality in one year. The median of factors among those who died was 3 (1-4), while it was 1 (0-2) among those who were alive at the end of the first year (p <0.001).

**Figure 2:** Number of unfavorable economic and psychosocial markers (EPSM) and their relationship with mortality in the first year after ICD implantation.

Table 2 shows the univariate comparison of clinical and psychosocial variables between individuals who died and survivors. In the multivariate analysis, including clinical factors that showed an association in the univariate analysis or with biological plausibility of association with the primary endpoint, only the presence of EPSM and LVEF were independent predictors of mortality in one year: HR 20.48 (2.75 – 52.29); p=0.003 and HR 0.97 (0.93 – 0.99); p=0.047, respectively (Table 3).

**Table 2:** Comparison of baseline characteristics of surviving individuals and those who died in the first year after implantation

	Survivors	Deaths	p
	167 (80.3%)	41 (19.7%)	
Male; n (%)	112 (67.1%)	31 (75.6%)	0.349
Age, average (±SD)	57 (±12)	62 (± 12)	0.040
Chagas cardiomyopathy, n (%)	86 (51.5%)	21 (51.2%)	0.999
Ischemic cardiomyopathy, n (%)	40 (24%)	12 (29.3%)	0.546
Other known etiologies, n (%)	52 (25.5%)	10 (19.2%)	0.307
Systemic arterial hypertension, n (%)	123 (73.7%)	30 (73.2%)	0.999
Diabetes, n (%)	38 (22.8%)	14 (34.1%)	0.159
Atrial fibrillation, n (%)	37 (22.2%)	14 (34.1%)	0.155
Previous stroke n (%)	21 (12.6%)	8 (19.5%)	0.312
Previous myocardial infarction, n (%)	47 (28.1%)	19 (46.3%)	0.038
Chronic kidney disease, n (%)	26 (15.1%)	10 (24.4%)	0.247
LVEF %, mean (± SD)	32.8 (± 9.5)	28.9 (± 8.2)	0.013
NYHA III, n (%)	64 (38.3%)	26 (63.4%)	0.005
MAGGIC score (% risk in 1 year), mean (± SD)	14.4 (± 9.1)	22.2 (± 13.2)	0.001
Socioeconomic vulnerability, n (%)	70 (41.9%)	32 (78.0%)	<0.001
Self-care deficit, n (%)	59 (35.3%)	24 (58.5%)	0.008
Poor pharmacological adherence, n (%)	31 (18.6%)	16 (39.0%)	0.011
Mood disorders, n (%)	58 (34.7%)	24 (58.5%)	0.007
CRP in VF/VT	57 (34,1%)	16 (39.0%)	0.586

LVEF: left ventricular ejection fraction; NYHA: New York Heart Association. Mood disorders: anxiety and/or depression; Self-care deficit: Self-care < 70 points; Low pharmacological adherence: Morisky < 6 points.

**Table 3:** Univariate and multivariate analysis of mortality less than one year after ICD implantation

	Analysis	p	Analysis	p
	Univariate		Multivariate	
	RR (95% CI)		HR (95% CI)	
Presence of EPSM	1.44 (1.29 – 1.61)	< 0.001	20.48 (2.75 – 52.29)	0.003
LVEF	0.96 (0.95 – 0.97)	<0.001	0.97 (0.93 – 0.99)	0.047
Chagas disease	1.01 (0.51 – 2.00)	0.999	1.12 (0.52 – 2.40)	0.773
NYHA III	2.27 (1.28 – 4.03)	0.005	0.99 (0.51 – 1.94)	0.978
Age	0.98 (0.98 – 0.99)	<0.001	1.01 (0.98 – 1.04)	0.423
Previous AMI	1.86 (1.08 – 3.19)	0.038	1.86 (0.85 – 4.05)	0.120

EPSM: economic and psychosocial markers; LVEF: left ventricular ejection fraction; AMI: acute myocardial infarction.

## Discussion

Psychosocial and socioeconomic factors were independently associated with higher mortality in the population of patients with HF undergoing ICD implantation for secondary prevention of sudden death. This demonstrates that alongside the clinical frailty caused by the disease, those with low pharmacological adherence, self-care deficits, socioeconomic vulnerability and mood disorders, EPSM, are more likely to die early.

Few studies in the literature have explored the correlation between psychological and socioeconomic factors and the prognosis of individuals with HF or those considered for high-cost CIED implantation.^4–10,19^

The strategy for assessing candidates for CIED implantation, or at least its structured approach to decision-making, is groundbreaking in Brazil and unprecedented globally for CIED cases. Thus, the MDT was essential for identifying important predictors that were determinants of mortality within our 12-month follow-up period. A similar model is implemented at a hospital in the United Kingdom, where 80% of cancer treatments are determined following consultations involving professionals from diverse health disciplines.^20^

The association between early mortality and EPSM likely arises from various mechanisms, notably patients’ limited understanding of disease progression, lack of autonomy or self-care capability, and challenges in medication adherence compounded by difficulties accessing healthcare resources.^10,11,18^

Deficits in self-care involve inadequate management practices and proactive measures regarding fluid and salt intake reduction, irregular medication use, smoking cessation, pneumonia and influenza vaccination, and systematic weight monitoring. Studies indicate that self-care practices are often inadequate among HF patients, influenced by sociodemographic, economic, cultural, educational, and etiological factors, and varying among different nationalities.^6,21,22^

It is estimated that around 60% of patients with HF have some degree of anxiety and/or depression, with only one-third of them being diagnosed.^4,5^ These patients have high rates of rehospitalization and early mortality, in addition to experiencing feelings, sensations, and events that affect their quality of life, such as difficulties adapting to the device, greater chances of infection, and inappropriate shocks.^4,23^ Our rate of patients identified with mood disorders was 39.8%, and they had an impact on mortality (p=0.012).

The rates of patients with inappropriate or non-systematic use of medications range from 30-60%. It is a multifactorial problem associated with polypharmacy, socioeconomic difficulties, intolerance, and access to drugs.^7^ A study with 557 patients evaluated the impact of medication adherence in patients with HF, one of the endpoints being death from any cause. After 1.1 years of follow-up, mortality was 28.5%, and those with low adherence were associated with mortality alone and with a two-fold increased risk of death, p<0.001.^24^ In our sample, despite being the marker with the lowest prevalence – 47/208 (22.5%), it was associated with mortality in the first year of the implant (p=0.011).

A relevant finding was the high prevalence of individuals with cardiomyopathy due to Chagas Disease. This fact could be related to a population with greater psychosocial vulnerability. However, the diagnosis of Chagas cardiomyopathy was not associated with higher mortality in the univariate or multivariate analysis. Therefore, this study raises the hypothesis whether the increased morbidity and mortality rates associated with cardiopathy due to Chagas Disease stems from a biological factor linked to infection and its mechanisms of myocardial damage, or if it reflects the poorer psychosocial conditions faced by these individuals since Chagas Disease predominates in low-income populations with inadequate sanitary conditions due to its mode of transmission.^3,10,17^

The one-year mortality estimated by the Maggic score was approximately 15%; however, the observed mortality during this period was 25%. Conversely, no deaths occurred within the one-year period among individuals without unfavorable economic and social markers. Thus, the elevated mortality rate in this population may be attributed to the impact of these markers, prevalent among those reliant on the public health system, where such markers are more prevalent. A study by Yusuf et al. indicates that socioeconomic factors exert a greater influence on the morbidity and mortality of cardiovascular diseases as socioeconomic status declines in the population.^25^

Limitations of the study include its single-center nature and the inclusion of a population with a high prevalence of unfavorable socioeconomic markers. However, this population is typically served by a reference service of the unified health system.

Therefore, we demonstrate that both psychosocial and socioeconomic factors are interconnected and have a significant impact comparable to factors directly related to the biology of the disease. Consequently, it is important to emphasize the need to conduct a multidisciplinary assessment focused on identifying other potentially modifiable issues, such as psychological disorders, deficits in self-care, and medication adherence, prior to ICD implantation, particularly in public health systems facing resource constraints. In addition, it is important to invest in factors not directly related to health, but which impact the endpoints of health policies.

## Conclusion

The presence of unfavorable psychosocial and economic factors among individuals with HF undergoing ICD implantation for secondary prophylaxis against sudden death was associated with increased mortality within the first year, indicating implants considered inappropriate by current guidelines.

Therefore, the inclusion of a MDT in the evaluation and discussions of the Device Team should be prioritized.
